# Condensation Mechanism of Hydrocarbon Field Formation

**DOI:** 10.1038/s41598-017-10585-7

**Published:** 2017-08-31

**Authors:** Oleg Batalin, Nailya Vafina

**Affiliations:** Oil and Gas Research Institute, Russian Academy of Sciences, Moscow, Russia

## Abstract

Petroleum geology explains how hydrocarbon fluids are generated, but there is a lack of understanding regarding how oil is expelled from source rocks and migrates to a reservoir. To clarify the process, the multi-layer Urengoy field in Western Siberia was investigated. Based on this example, we have identified an alternative mechanism of hydrocarbon field formation, in which oil and gas accumulations result from the phase separation of an upward hydrocarbon flow. There is evidence that the flow is generated by the gases released by secondary kerogen destruction. This study demonstrates that oil components are carried by the gas flow and that when the flow reaches a low-pressure zone, it condenses into a liquid with real oil properties. The transportation of oil components in the gas flow provides a natural explanation for the unresolved issues of petroleum geology concerning the migration process. The condensation mechanism can be considered as the main process of oil field formation.

## Introduction

Petroleum geology successfully explains how hydrocarbon fluids are generated during sedimentation but gives no clear explanation regarding further processes, such as how fluids are expelled from source rocks (primary migration) and move to reservoirs (secondary migration)^1, 2^. Hydrocarbons are assumed to leave a kerogen matrix via diffusion and enter the pore space of source rocks. Then, as was concluded from a long-lasting discussion of various possibilities, the primary migration occurs in the form of a hydrocarbon phase that is separated from the water^3–5^. However, it is unclear why this process occurs and what factors drive the process. In particular, kerogen yields contain copious asphaltenes and resins throughout almost the entire course of destruction^6^. Such a liquid has difficulties to migrate due to high viscosity and strong adhesion to rocks. To add to these hurdles, source rocks usually have low permeability. A variety of methods to explain the migration mechanism have been proposed^3, 4, 7, 8^, including rock microfracturing^9, 10^. Furthermore, the special role of water in oil formation and expulsion has been studied^11^. Despite the particular importance of the primary migration, its key mechanism is unknown; therefore, in practice, overly simplistic models are used^12^.

Secondary migration has been experimentally^13, 14^ and theoretically^1, 15^ studied. It is assumed that oil migration occurs due to buoyancy forces along restricted pathways at first vertically and then by migration below a sealed surface to a trap location. The migration pathways are difficult to determine; thus, they are often inferred based on indirect signs such as molecular markers/indices^16–18^, oil-bearing fluid inclusions^19^, or a set of these types of indicators^20^. The pathways are also determined based on the morphology of the sealing surfaces, which enable us to predict the potential localization of hydrocarbon accumulation with a certain probability^21^. Nevertheless, the process of oil migration from the suspected source rock to the reservoir has remained elusive.

When studying migration, it is explicitly or implicitly assumed that oil moves in the liquid phase. However, there is an alternative possibility: the transfer of oil components in the gas phase. In the 1960s, it was experimentally shown that high-pressure gases are good solvents for oil hydrocarbons^22^. At moderate pressure, heavy oil components are poorly soluble in gas, and this fact was used as an argument against oil being carried in a gas phase^23^. Nevertheless, in the more severe pressure-temperature conditions that exist at depths of 5 km or deeper, the solubility increases to a sufficient level^24^. The gases produced in the deep part of the basin are capable of effectively capturing oil hydrocarbons from source rocks and carrying them to shallower depths, where they condense^25^. There is only one example^26^ of oil accumulation (“light oil”) that is formed by condensation from a migrating gas. However, this example cannot be considered as valid. This “light oil” widely differs from normal oil because of its unusually low molecular weight and C_10+_ density^26^. Thus, the possibility of forming real oil fields due to the transport of oil components in gas remains a hypothesis based only on laboratory experiments.

To identify the mechanism underlying migration and hydrocarbon field formation, we selected the multi-layer Urengoy field, in which a large amount of data is available. It is necessary to clarify whether gases generated at the bottom of a sedimentary basin can carry oil components to shallower depths. This study consists of two parts. In the first part, the generation and subsequent behavior of such gases are traced. The second part investigates the formation mechanism of Urengoy oil and gas accumulations to determine whether their formation is a result of hydrocarbon flow arriving from great depths.

## Results

### An upward hydrocarbon flow

The bulk of the gas is generated via the destruction of oil hydrocarbons that are formed earlier during kerogen craсking (secondary destruction gases). It is possible to trace the stages of upward gas flow formation based on well-understood physical phenomena. In sedimentary basins, due to the compaction disequilibrium and hydrocarbon fluid generation^1, 27^, a pore pressure in excess of the hydrostatic pressure is observed at a depth greater than 3 km^28^. Model calculations indicate that at the stage of kerogen conversion into oil-type hydrocarbons, the pore pressure can approach the lithostatic pressure only at unusually low permeability conditions^10, 29, 30^. In the next step, during the transformation of oil hydrocarbons into gas (secondary destruction), the pore pressure rapidly increases to the lithostatic pressure^31–33^. When the pressure reaches 0.7–0.9 times the lithostatic pressure (equal to 1.6–2.1 times the hydrostatic pressure), microfractures appear. These microfractures grow and merge, forming a connected fracture system. The fracture-type permeability facilitates the primary migration^34^. Under such circumstances, an effective mechanism of hydrocarbon transfer appears, which is caused by the buoyancy-driven propagation of isolated fluid-filled fractions^35^. The gases of secondary destruction are characterized by high pressure, and the oil components easily dissolve in these gases. Thus, an upward flow that transports the oil hydrocarbons is formed. The flow predominantly captures light oil components^36^, which explains why the fractions of asphaltenes and resins in oil fields are much lower than those in kerogen yields. When the upward fluid flow reaches confining rocks, the pressure begins to grow until these rocks are broken through^28^. The simulation results^37^ indicate that a large quantity of hydrocarbons may pass through fractures in the cap rocks in a short time.

**Figure 1:**
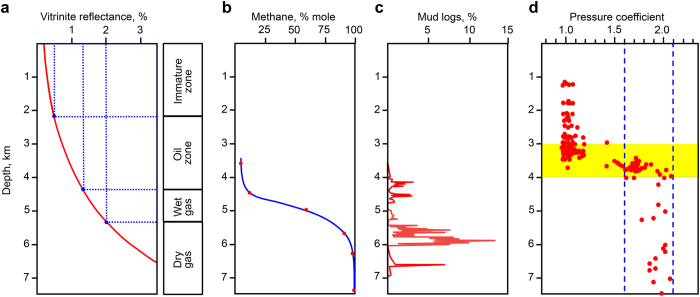
Evidence for upward flow in the Urengoy region. (**a**) Vitrinite reflectance and the corresponding zones of hydrocarbon fluid generation. (**b**) Methane molar fraction in the pore-space gas (SG-7)^38^. (**c**) Gas escape peaks on mud logs (SG-6)^39^. (**d**) Pressure coefficient K_p_ of the northern Western Siberia fields^40^. K_p_ values (circles) up to the Upper Jurassic–Lower Cretaceous regional seal (3 to 4 km) fall into the fracturing pressure range of 1.6–2.1, which indicates migration processes.

Such an upward flow formation can be observed in the Urengoy field. To illustrate this process, data from “ultra-deep” wells within a group of fields called the Greater Urengoy Area were used. The Tyumen (SG-6) and En-Yakha (SG-7) wells were included, which are drilled to 7502 m and 8250 m, respectively. Well SG-6 is located in the center of the Koltogor-Urengoy rift, which is 60 km east of Urengoy. Well SG-7 is between the Pestsovoye and Yen-Yakha fields. According to the standard scheme of oil and gas generation based on the vitrinite reflectance, the gas zone in this region begins at 4.3 km (Fig. 1a). At a greater depth, the proportion of methane in the gas increases (Fig. 1b, Well SG-7). The gas content in the pore space reaches the highest values in the range of 4.5–6.5 km, where it exceeds 50 cm^3^/kg (Well SG-7^38^). Isotopically light methane with δ^13^C_CH4_ from −45 to −75 pointing to oil component destruction is observed at a depth of 5.5–8 km (Well SG-7^41^). According to mud logging data, gas liberation occurs in a large depth interval with a maximum value at 5.7–6.0 km (Fig. 1c, Well SG-6^39^). Hydraulically induced microfractures have been observed in Well SG-6^42^ with a maximum amount in the interval of 5100–5650 m. Core samples from a depth of 5663–5685 m in Well SG-7 exhibit horizontal microfractures with an aperture of approximately 5 micrometers. Based on these data, the upward flow is apparently generated at a depth of 5–6 km.

High-pressure gases dissolve oil components at the flow generation moment and during the upward migration through rocks (for example, through the Bazhenov formation) with a high content of liquid hydrocarbons previously generated. The upward flow transfers the high pressure from deeper to shallower formations (see example in ref. 43). Thus, in a large interval up to the regionally extended Upper Jurassic–Lower Cretaceous seal (located at a depth of 3–4 km) at a pressure coefficient K_p_, the pore pressure-hydrostatic pressure ratio remains high^40^, i.e., 1.6–2.1 (see Fig. 1d). This value is exactly within the range at which fractures appear and thus provides the possibility for migration. When sufficient energy is accumulated, the fluid breaks through the confining rocks and enters overlying reservoirs.

Thus, all these data indicate that the generation of secondary destruction gases leads to the formation of an upward flow capable of transferring oil components to shallower depths. In Urengoy, there is evidence that flow generation occurs at a depth of 5–6 km.

### Formation of the Urengoy field

The Urengoy field^44, 45^ is one of the three largest gas fields in the world. Its hydrocarbon in-place volumes are 16 TCM of gas and 1.2 billion tons of gas condensate. The field is located at the Urengoy megaswell, which is a N-S extended anticline. The field was discovered in 1966. Later, the Yen-Yakhinskaya, Tab-Yakhinskaya areas, Pestsovoye and a number of other fields were discovered in the vicinity, which together with the Urengoy field have been named Greater Urengoy, which covers a total area of about 6,000 km^2^. The main pay zones are in Cenomanian (1030–1260 m), Neocomian (1700–3100 m), Achimov (3500–4000 m), and Jurassic reservoirs. The Paleozoic basement is at a 5–7 km depth.

- The *Cenomanian reservoir* (methane 98.5%, nitrogen 1%) contains the main in-place volumes of gas of the Urengoy field. The principal source of gas is a methane release from groundwater due to pressure reduction during the Cenozoic uplift^46^. High reservoir permeability of 1000 mD ensured high gas flow rates of the production wells. In the late 1980s, the Cenomanian reservoir provided a half of the country’s gas production, 300 billion m^3^ of gas per year.
- *The Neocomian* productive complex contains 22 deposits (1700 m to 3100 m). The reservoirs are represented by alternating sandstones, siltstones and mudstones. There are only gas-condensate (GC) reservoirs without noticeable oil rims at a depth shallower than 2500 m; whereas the deeper reservoirs have oil rims (GC + O). All the gas-condensate fluids are saturated^47^. Initial in-place volume of oil in the oil rims in BU_8_-BU_14_ reservoirs is 300 million tons. Thermogenic gases coming from the depth 4–8 km are considered to be the primary source of hydrocarbons accumulated in the Neocomian reservoirs^48^, and at the same time methane released from water is gradually added to the reservoir fluids at a shallower depth.
- The *Achimov reservoirs* (containing both gas-condensate fluid and oil) are saturated by hydrocarbons within the area of 11,000 km^2^. Most of hydrocarbons in place within this area belong to Greater Urengoy (4 trillion m^3^ of gas and 2 billion tons of liquid hydrocarbons). The Achimov clinoform complex within the confines of the Urengoy field includes 6 reservoirs, Ach_3–4_ and Ach_5_ being the most significant ones. The Achimov reservoirs are overpressured. The sedimentary rocks in the base part of the Koltogor-Urengoy rift^49^ are considered to be a source of hydrocarbon fluids in the Achimov reservoirs.
- The *Jurassic reservoirs* are represented by alternating sandstones, siltstones and mudstones, which are distributed unevenly. Reservoir porosity is 14–15%. Pressure ratio is 1.6–1.95.

Characteristics of the main Neocomian and Achimov reservoirs are summarized in Table 1. The neighboring reservoirs, which are often hydrodynamically connected, were combined into clusters from G_1_ to G_8_. For each cluster, the average depth, temperature and pressure were determined (see Table 1).

**Table 1:** Characteristics of Urengoy reservoirs.

Сluster	Reservoir	Type of fluids	Depth, m	Temperature, °С	Pressure, MPa
G_1_	AU_9–10_	GC	2100	62	21
G_2_	BU_1–2_	GC	2300	65	23.4
G_3_	BU_5–6_	GC	2450	70	25.5
G_4_	BU_8_^0^	GC + O	2620	76	27.5
	BU_8_	GC + O			
	BU_9_	GC + O			
G_5_	BU_10_	GC + O	2750	79	28.3
	BU_10_^0^	GC + O			
	BU_11_	GC + O			
G_6_	BU_12_	GC + O	2850	80	29.5
G_7_	BU_13_	GC + O	3000	85	32
	BU_14_	GC + O			
G_8_	Ach_3–4_, Ach_5_	GC + O	3650	105	60.5

To show that the Urengoy field is formed as a result of the liquid-gas phase separation of upward flow, a phase behavior simulation was conducted. We consider the following model of hydrocarbon field formation.

1. After breaking through the Upper Jurassic–Lower Cretaceous seal (depth from 3 to 4 km), the upward hydrocarbon flow simultaneously enters several neighboring reservoirs (G_7_, perhaps more) at depths of less than 3 km, where the fluid separates into gas and liquid phases under *in situ* temperatures and pressures. Then, gravitational segregation occurs. Gas occupies the upper part of the trap, forming a gas cap, and the liquid forms an oil rim at the base.
2. Gas from the gas cap gradually migrates through the cap rocks into the next shallower trap, where some liquid may condense. Then, in a similar manner, the gas successively passes through the overlying traps G_6_, G_5_, G_4_… one above another.

Because the exact composition of hydrocarbon flow in the place of its origin is unknown, the composition of gas-condensate fluid from G_8_ – the deepest Urengoy cluster of reservoirs (average depth: 3650 m) – was taken as the initial composition. Using this composition, the phase separation was calculated for an instantaneous fluid charging neighboring reservoirs (see No. 1) and the sequential passage of the gas flow through the overlying traps (see No. 2).

**Figure 2:**
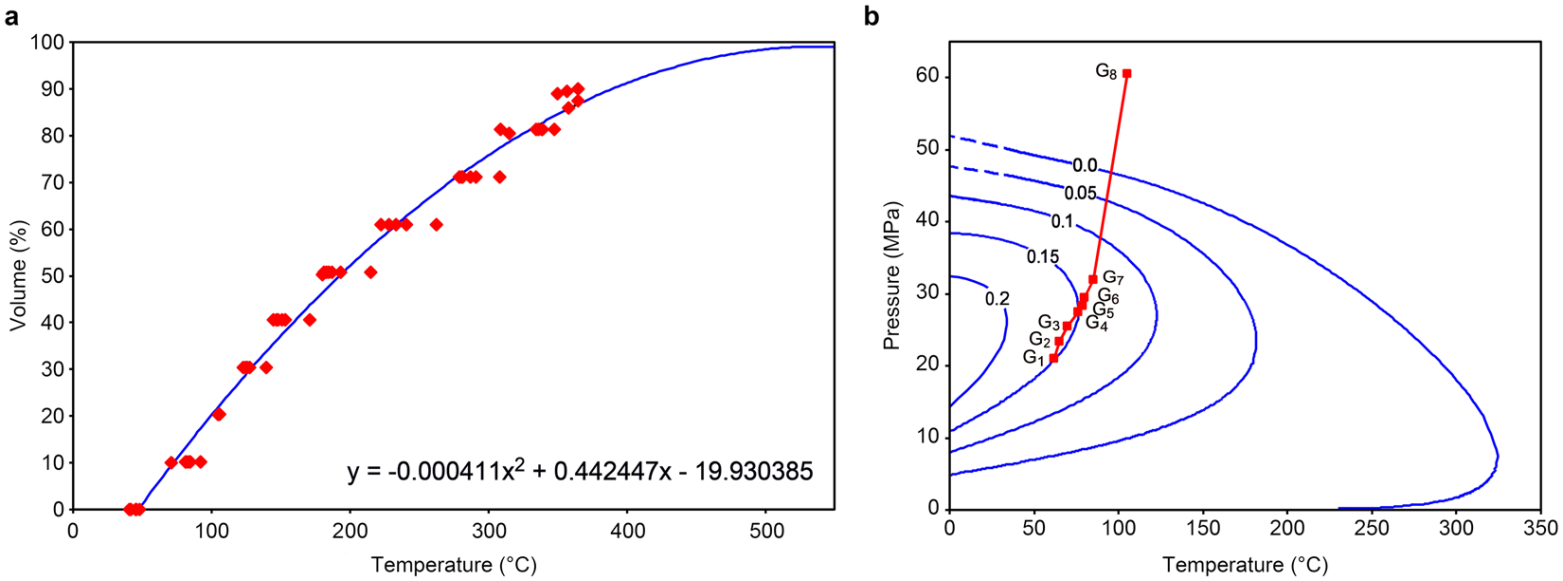
Starting characteristics of upward flow. (**a**) Approximate curve (second-degree polynomial) for G_8_ DBC distillation. Diamonds – distillation data for different wells^47, 50^. (**b**) Phase diagram of the G_8_ gas-condensate fluid and the upward fluid flow path on the P-T plane. The upward flow (polyline) passes through the G_8_, G_7_… G_1_ traps (squares). The curve numbers indicate the volume fraction of liquid that results from the phase separation of the G_8_ fluid under these PVT conditions.

The composition of gas-condensate fluid in G_8_ (in mol%) is С_1_ = 78.35, С_2_ = 8.6, С_3_ = 3.8, С_4_ = 1.68, CO_2_ = 0.1, N_2_ = 0.81, С_5+_  = 6.66^47^. Molar quantities (Table 2, column 3) of the lumped fractions with pre-defined boiling point ranges (Table 2, column 2) were determined from the debutanized condensate (DBC) distillation curve (Fig. 2a), generated by averaging the data from several wells. However, such C_5+_ characterization for the G_8_ gas-condensate fluid would not be quite accurate due to the fact that the uncontrolled amounts of the lightest fractions had been carried away with the separated gases in the process of preparation of the DBC sample itself. The correction procedure described in “Меthods” was applied to account for this effect. The corrected molar quantities of the lumped fractions for of the G_8_ reservoir fluid are shown in Table 2, column 4.

**Table 2:** Lumped fractions content in the G_8_ reservoir fluid composition

Fraction	Range, ^о^С	Composition determined from DBC distillation, mol.%	Corrected composition, mol.%
F_1_	26–60	0.5892	0.7612
F_2_	60–95	1.2015	1.2116
F_3_	95–122	0.7932	0.7695
F_4_	122–150	0.7172	0.6908
F_5_	150–200	1.0764	1.0344
F_6_	200–250	0.8678	0.8335
F_7_	250–300	0.6016	0.5779
F_8_	300–350	0.3619	0.3476
F_9_	350–400	0.2343	0.2251
F_10_	400-…	0.2169	0.2083

The phase diagram, calculated based on the obtained fluid composition of G_8_ (gas components + fractions), is shown in Fig. 2b. The calculated dew point pressure is 46.215 MPa at a reservoir temperature 105 °С. The earlier PVT experiments^51^ indicated a dew point pressure of 46.0–46.4 MPa (for the most likely С_5+_ content of 350–400 g/m^3^ in the reservoir gas). Thus, the calculated result is in good agreement with the actual data, which indicates that the reservoir fluid model correctly describes its phase behavior. The pressure-temperature (P-T) conditions of reservoirs G_8_ to G_1_ are indicated in Fig. 2b. As shown, when a gas-condensate fluid of G_8_ reaches these conditions, it separates into gas and liquid phases.

Below are the results of our calculations in comparison with the actual data for the Urengoy field, namely, the characteristics of liquid and gas fluids obtained by simulating the phase separation of the upward flow at different depths in reservoirs G_7_… G_1_. Our main goal was to show how oil forms from an upward gas-condensate flow at shallower depths. There is a direct link between the composition / physical properties of any oil and its distillation curve. For this reason, comparison of the actual and calculated distillation curves for degassed oil and debutanized condensate has been chosen as a method to test and prove this oil formation concept.

### Formation of oil

**Figure 3:**
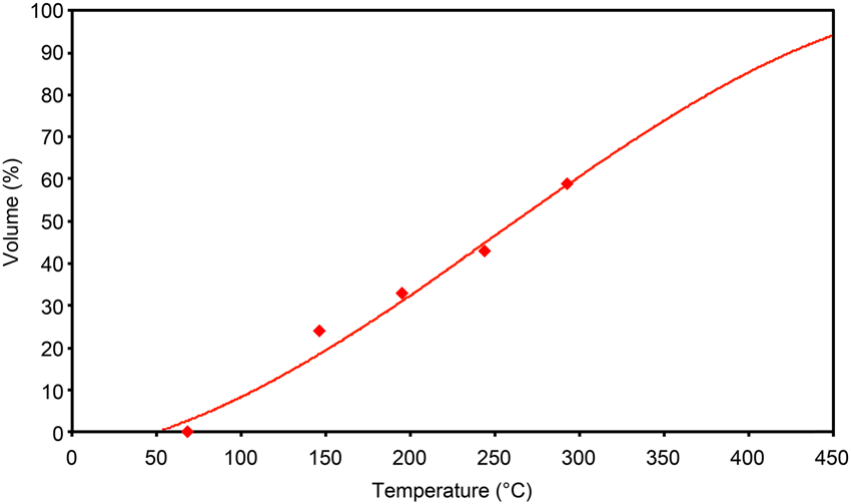
Oil formation from upward flow. Comparison of the calculated distillation of G_7_ degassed liquid (curve) with the actual distillation of BU_14_ (belongs to cluster G_7_) degassed oil (diamonds^47^).

The analysis of the main properties of the liquid in G_7_, which results from the phase separation of the upward flow, reveals that it is conventional oil. To demonstrate this fact, Fig. 3 shows the calculated results of the G_7_ degassed liquid distillation compared with those of the distillation of real BU_14_ oil (reservoir BU_14_ belongs to cluster G_7_). The results indicate good agreement between the theoretical curve and actual data. Additionally, the density and molecular mass show a similarity. The calculated density is 0.826 g/cm^3^; the real density is 0.837 g/cm^3^. Note that the density of primary oils (not exposed to secondary transformation) from northern Western Siberia^52^ is in the range of 0.830–0.838 g/cm^3^. The calculated molecular mass is 194; the actual molecular mass is 197.

### “Liquid components” content in the upward flow

**Figure 4:**
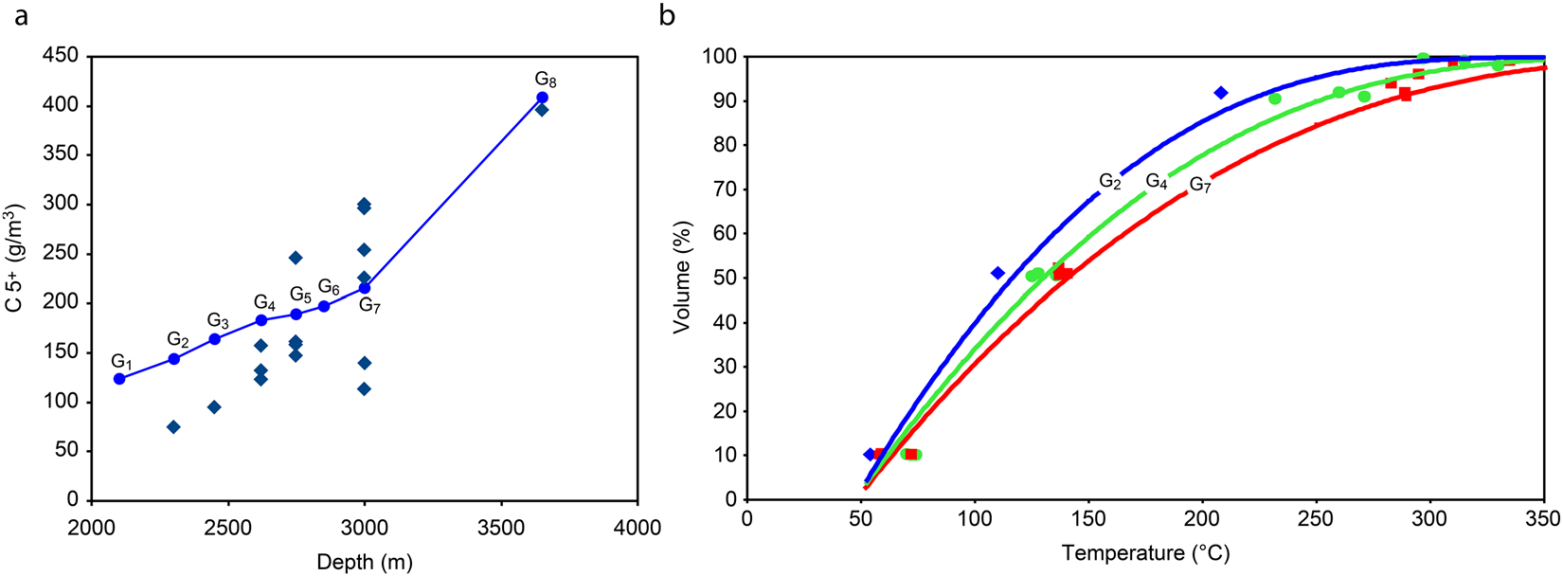
Properties of gas accumulations. (**a**) Calculated C_5+_ content of G_8_, G_7_… G_1_ gas accumulations formed at the phase separation of the upward flow (circles) and actual data (diamonds^47^). (**b**) Comparison of the calculated DBC distillation for G_2_, G_4_, and G_7_ reservoirs (blue, green, and red curves, respectively) and actual data^47^ (blue diamonds, green circles, and red squares, respectively).

Due to the pressure and temperature drop, a part of the “liquid components” (C_5+_) condenses from the upward flow. Higher pressure helps capture heavier components into the flow, in particular, heavy alkanes, as it was shown in ref. 36. Therefore, the heavy alkane content in oil may indicate the thermobaric conditions that existed upon departure of hydrocarbons from the source rock. Gas accumulations are formed, in which the proportion of “liquid” components gradually decreases. Figure 4a shows the calculated and measured C_5+_ content in the gas cap of G_8_, G_7_… G_1_.

### Genetic relationship of all the Urengoy fluids

Figure 4b shows DBC distillation curves calculated for G_7_, G_4_, and G_2_ reservoirs compared with the gas-condensate well test data^47^ (for simplicity, only the centers of measured ranges are shown, not the whole range of data). The portion of the heaviest fractions was consistently reduced at shallower depths. Accordingly, the end boiling temperature of DBC becomes lower. Based on the conformity between calculated and actual data, the genetic relationship of the Urengoy gas-condensate fluids G_7_ - > G_4_ - > G_2_ is clearly observed. All of them come from a common source: G_8_.

### The process of the reservoirs filling

**Figure 5:**
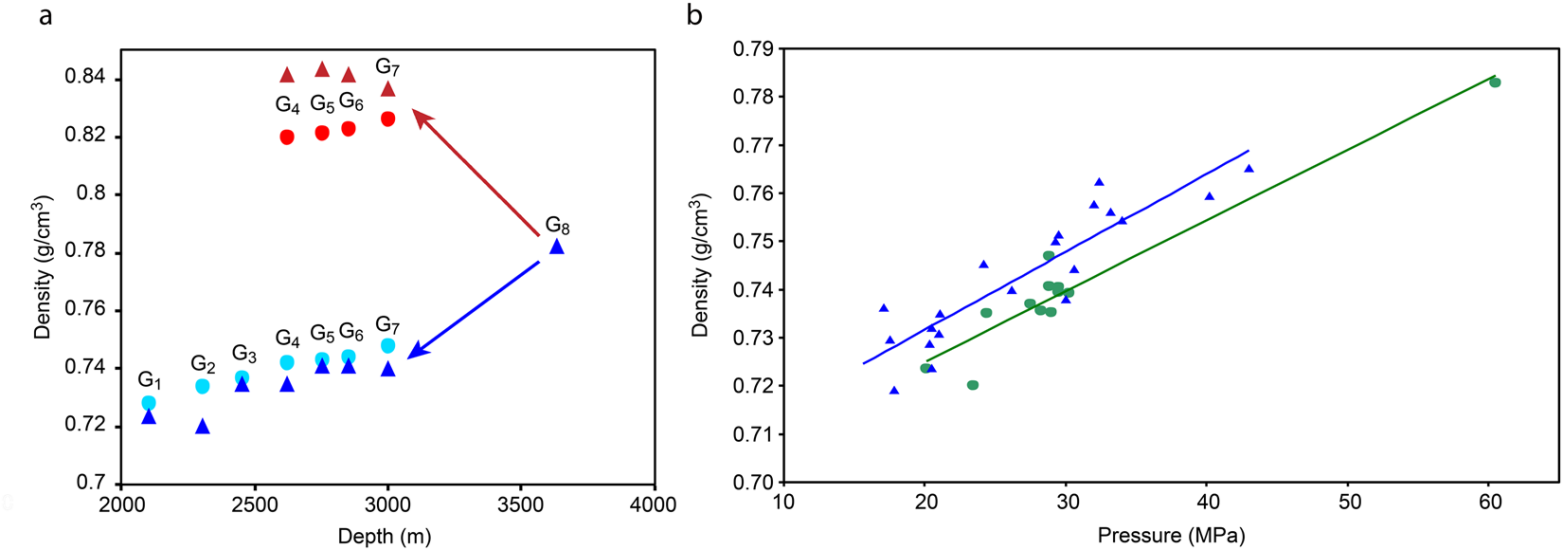
Fluid densities at different depths (pressures). (**a**) Illustration of reservoir filling. Oil rim formation via G_8_ gas-condensate fluid injection into adjacent traps G_7_, G_6_, G_5_, and G_4_ and subsequent gas migration through G_7_… G_1_ traps. Oil in G_7_, G_6_, G_5,_ and G_4_ traps: red circles – calculated degassed oil density; dark red triangles – actual data^47^. Gas accumulations in G_7_… G_1_ traps: blue circles – calculated DBC density; dark blue triangles – actual data^47^. (**b**) DBC density variation with reservoir pressure. Circles – Urengoy reservoirs^47^; triangles – other fields in northern Western Siberia^45^; straight lines – corresponding linear regressions.

Upon breaking through the Upper Jurassic–Lower Cretaceous regional seal, the G_8_ gas-condensate fluid simultaneously enters the shallower traps G_7_, G_6_, G_5_, and G_4_ under their different P-T conditions. Due to a significant pressure drop, a large quantity of liquid condenses from the G_8_ fluid in the above-mentioned reservoirs, thereby forming the oil rims of Urengoy. In the gas-condensate fluid of G_8_, several asphalt-resinous substances are concentrated in high-boiling (more than 450 °C) distillation fractions (see Fig. 2a). When such a fluid is injected into G_7_, G_6_, G_5_, or G_4_, the asphalt-resinous substances condense, which gives the liquid a black color typical of oil. The high-boiling fractions are absent in the gas phase of G_7_, G_6_, G_5_, and G_4_ (see Fig. 4b). Therefore, when this gas migrates upward, a lighter liquid condenses. Due to a small pressure drop at the jumps from one layer to another, a small quantity of liquid drops out. Thus, this process does not lead to oil creation and oil rim formation in G_3_, G_2_, and G_1_. It can be concluded that the Urengoy oil in G_7_, G_6_, G_5_, and G_4_ can only originate from a high-pressure fluid that is rich in heavy components and injected from a greater depth. All the shallower traps (G_3_, G_2_, G_1_) are filled with gas. The gas phase in all the traps from G_7_ to G_1_ follows the same trend and becomes lighter due to a gradual loss of heavy components during its upward migration. This trend can be quantified by the DBC density, which decreases from G_7_ to G_1_. Figure 5a shows densities of the degassed fluids (dead oil and DBC) of the reservoirs. Based on these densities, the splitting of the “liquid components” of G_8_ into two parts in the upper traps was observed (Fig. 5a). The heavier liquid components predominantly move to the oil, whereas the lighter ones mainly remain in the gas flow. The increase in the densities of the real oils G_6_, G_5_, and G_4_ relative to G_7_ oil is explained by the absence of light boiling fractions (F_1_, F_2_), possibly washed out by the gas flow.

The decrease in DBC density with decreasing depth (pressure) is observed in many fields. Figure 5b demonstrates a linear correlation between the DBC density and pressure for the Urengoy and other fields of northern Western Siberia, which indicates a similar formation.

The agreement between the predicted oil and gas properties and the actual data over the entire depth interval provides evidence that the Urengoy field is formed as a result of the phase separation of upward flow. Starting with the hydrocarbon flow composition at a certain depth, it is possible to predict the composition of oil and gas in shallower reservoirs through the simulation of phase transitions. Simulated composition and physical properties of the liquid correspond to oil. The composition of gas accumulations formed by the upward flow becomes gradually lighter due to the precipitation of heavy components.

## Methods

The simulation of phase behavior was based on the Peng-Robinson equation of state. The composition of hydrocarbon fluids is specified by the indication of molar fractions of individual components (methane, ethane, propane, butane, non-hydrocarbon gases) and lumped fractions (or pseudo-components). We address the phase behavior of fluids (gas, condensate, oil) at a high pressure in a reservoir and at a low pressure at standard conditions (20 °С, 1 atm). There are a wide variety of fluids whose distillation fraction properties are significantly different. However, to perform the calculations, fixed fractions must be set for all fluid types. The best method to take into account all the features of the fluids is the identification of “hybrid fractions.” At low boiling temperatures (up to 250 °С), the characteristics of “hybrid fractions” are based on the condensate distillation (“condensate fractions”); at higher temperatures, they are defined in a conventional manner (“standard fractions”) – see Table 3. To calculate the density of liquids under standard conditions, we assumed that the liquid’s volume equals the sum of the volumes of all components. Hence, the “condensate fractions” were used for the condensate density calculation, whereas the “standard fractions” were used for the density of dead oil.

**Table 3:** Characteristics of fluid fractions.

Fraction	Temperature, °С	Condensate fractions		Standard fractions		Hybrid fractions	
		Density, g/cm^3^	Molecular mass	Density, g/cm^3^	Molecular mass	Density, g/cm^3^	Molecular mass
F_1_	43	0.6213	68.71	0.6608	78.41	0.6213	68.71
F_2_	77.5	0.7072	98.98	0.7083	91.57	0.7072	98.98
F_3_	108.5	0.7336	111.89	0.7374	103.36	0.7336	111.89
F_4_	136	0.7539	123.44	0.7594	117.60	0.7539	123.44
F_5_	175	0.7729	135.96	0.7827	139.59	0.7729	135.96
F_6_	225	0.7895	148.52	0.8097	173.37	0.7895	148.52
F_7_	275	0.8015	158.75	0.8357	214.47	0.8186	186.61
F_8_	325	0.8106	167.26	0.8570	263.00	0.8570	263.00
F_9_	375	0.8180	174.73	0.8770	318.00	0.8770	318.00
F_10_	450	0.8248	182.07	0.975	500	0.975	500

In the Urengoy field, the G_8_ gas-condensate composition was determined based on fluid samples taken from a surface separator. Gas components were determined on a chromatograph, the multi-component “lumped” fractions of С_5+_ were determined from the debutanized condensate (DBC) distillation. DBC was obtained in the following process. The wellstream was separated at 40–50 MPa in a separator into gas and crude condensate; the latter was then stabilized at atmospheric pressure; then the components lighter than pentane were removed from the degassed condensate; and finally, DBC was recovered. In the process of separation, degassing and debutanization, some light fractions were carried away by separating gases in an uncontrolled manner adding an error to calculation of the original G_8_ composition.

To address this issue, a computer simulation^53^ of the phase separation process in the following chain: gas-condensate fluid in reservoir - > condensate in separator - > degassed condensate - > DBC was used. Based on this model, the inverse problem was numerically solved. As the result, the original G_8_ composition (by individual gas components and fractions) was back-calculated from the known composition of DBC.

## Discussion

The transfer of oil components by the gas flow naturally explains primary and secondary migration. Difficulties with primary migration arise if it is assumed that oil migrates as a liquid from the beginning. In this case, there are unresolved issues regarding driving forces, a high content of asphaltene-resinous components and residual saturation. If we assume that oil components migrate in the gas phase, there are no such difficulties. The generation of secondary destruction gases leads to high pressure, which creates fracturing conditions in source rocks. Under such high pressure, the oil components become soluble in gas. The gas, when generated, directly contacts the liquid products of kerogen destruction and, therefore, quickly becomes highly saturated with oil hydrocarbons. The migration of gas together with oil components through microcracks (primary migration) and secondary migration in rocks or along the cracks is not a problem because of high gas mobility. The transfer of oil components occurs in the gas phase; therefore, oil does not migrate as a liquid. Accordingly, there are no traces of residual oil saturation. Oil migrates as a liquid only after it has condensed from the flow. Oil field formation begins with the condensation of microdroplets of oil from the upward flow. Then, oil concentrates at the bottom and forms an oil rim. While charging a trap, the oil rim moves downwards and is pushed by the increasing volume of gas until the trap is completely filled. Then, bulk phase oil begins to migrate laterally and upward, which charges adjacent traps.

A hydrocarbon flow that transports oil hydrocarbons from a greater depth upwards necessarily originates in the sedimentary basins capable of generating secondary destruction gases. So, oil fields can be formed by this way, when a sedimentary basin is sufficiently deep to generate the secondary destruction gases. It is known, that the sedimentary basins with large oil reserves have a depth of more than 5 km; all large and especially giant fields belong to deep sedimentary basins^54^.

Once emerged, the upward flow will continuously progress. The flow intensity depends on the amount of organic matter in the rocks that enter the flow generation zone. Thus, a large quantity of oil accumulates at the depth of condensation over geological time. The depth at which the upward flow originates is defined as 5–7 km or greater. Here, with the pressure ratio of 1.6–2.1, the pore pressure is 80–140 MPa. At such a high pressure, the gas saturation with oil hydrocarbons is approximately 1 kg/kg or more^7^. Specifically, the gas carries an equal mass of oil hydrocarbons. Only a small part of the gas from the flow is retained in the traps, whereas the oil condensed from the flow remains. Therefore, the oil mass brought by the flow into the oil deposits is many times greater than the mass of all gas accumulations existing in the basin. These arguments allow us to suggest that the transposition of oil hydrocarbons by gas provides the bulk of oil in the oil fields.

The oil- and gas-bearing basins are the results of consecutive stages in one evolutionary process. Initially, gas accumulations with small oil rims appear. Then, the traps are gradually filled with new portions of oil that fall out from the upward flow, whereas gas escapes through the cap rock. When the hydrocarbon flow stops and all gas escapes, only oil fields remain in the basin.

## Conclusions

We have identified an alternative mechanism for hydrocarbon field formation, which is that oil and gas accumulations are formed by phase separation of an upward hydrocarbon flow. At the stage of secondary kerogen destruction, a large quantity of gas is produced to create high pressure and generate microfractures. An upward gas flow arises. The flow captures dispersed oil hydrocarbons, which have no other path to exit the source rock. Oil components are carried by the gas flow, and when the flow reaches a low-pressure zone, part of it condenses into liquid that has the properties of real oil. With decreasing depth, the fluids of gas-condensate accumulations formed by upward flow gradually become lighter because of the loss of the heaviest fractions.

Oil, as a liquid with a well-known composition and properties, appears at the moment of its condensation from the hydrocarbon flow. Two main steps of transformation can be determined. First, oil hydrocarbons that are dispersed in source rocks dissolve in the gas flow, predominantly the lighter ones. The second step is related to the condensation from the flow. Preferably, the heavier fractions condense into the oil.

The transportation of oil components in the gas flow explains the unresolved issues of petroleum geology concerning the mechanism of migration. The natural origin of an upward hydrocarbon flow in sufficiently deep sedimentary basins and its sustained activity over geological time lead to large oil accumulations at the depth of condensation. These factors provide a basis to consider the transportation of oil components in a gas flow and subsequent condensation as a main process of oil field formation.
